# Iodine Deficiency Exacerbates Thyroidal and Neurological Effects of Developmental Perchlorate Exposure in the Neonatal and Adult Rat

**DOI:** 10.3390/toxics12120842

**Published:** 2024-11-23

**Authors:** Mary E. Gilbert, MaryAnn G. Hawks, Kiersten S. Bell, Wendy Oshiro, Carmen Wood, Barbara Jane George, Ryne Thomas, Jermaine Ford

**Affiliations:** 1 Centre for Public Health and Environmental Assessment, Office of Research and Development, US Environmental Protection Agency, Research Triangle Park, NC 27709, USA; hawks.maryann@epa.gov (M.G.H.); oshiro.wendy@epa.gov (W.O.); wood.carmen@epa.gov (C.W.); thomas.ryne@epa.gov (R.T.); 2 Oak Ridge Institute for Science and Education, Oak Ridge, TN 37830, USA; kierstenbell@utexas.edu; 3 Division of Pharmacology and Toxicology, College of Pharmacy, University of Texas, Austin, TX 78712, USA; 4 National Center for Computational Toxicology, Office of Research and Development, US Environmental Protection Agency, Research Triangle Park, NC 27709, USA; ford.jermaine@epa.gov

**Keywords:** perchlorate, brain, development, neurotoxicity, thyroid hormone, iodine deficiency

## Abstract

Thyroid hormones (THs) require iodine for biosynthesis and play critical roles in brain development. Perchlorate is an environmental contaminant that reduces serum THs by blocking the uptake of iodine from the blood to the thyroid gland. Using a pregnant rodent model, we examined the impact of maternal exposure to perchlorate under conditions of dietary iodine deficiency (ID) on the brain and behavior of offspring. We observed modest reductions in thyroxine (T4) in the serum of dams and no effect on T4 in pup serum in response to maternal exposure to 300 ppm of perchlorate in the drinking water. Likewise, serum T4 was reduced in ID dams, but, as with perchlorate, no effects were evident in the pup. However, when ID was coupled with perchlorate, reductions in pup serum THs and transcriptional alterations in the thyroid gland and pup brain were detected. These observations were accompanied by reductions in the number of cortical inhibitory interneurons containing the calcium-binding protein parvalbumin (Pvalb). Alterations in Pvalb expression in the neonatal brain were associated with deficits in the prepulse inhibition of acoustic startle in adult male offspring and enhanced fear conditioning in females. These findings support and extend structural defects in the brain previously reported in this model. Further, they underscore the critical need to consider additional non-chemical stressors in the determination of hazards and risks posed by environmental contaminants that affect the thyroid system.

## 1. Introduction

Neurodevelopmental disorders can arise due to changes in the developmental trajectories of neurons during the early stages of circuit assembly in the brain. Thyroid hormones (THs) play a critical role in orchestrating the timing of those trajectories. Iodine is a fundamental element required for TH synthesis. Iodine deficiency occurs in many regions around the globe and is of substantial concern for pregnant women, when THs increase dramatically to meet the needs of the developing fetus [1,2,3]. Severe maternal iodine deficiency has devastating effects on fetal growth and development, resulting in permanent deficits in brain development and function [4,5,6,7,8]. Despite the success of universal salt supplementation programs, iodine-deficient (ID) regions remain throughout the world and are particularly prevalent in countries reliant on desalinated seawater as a source of safe drinking water [9,10,11,12]. Within the US, although some pockets of moderate deficiencies may remain in the northern regions, the more recent concern is an increasing trend towards marginal iodine deficiency in the general population, attributed largely to dietary changes and the reliance on more processed foods. Alarmingly, this trend is also prevalent in women of childbearing age [6,13,14].

The sodium iodide symporter (NIS) is a transporter protein that actively shuttles iodide across mammalian intestinal and thyroid epithelia to supply iodine for thyroid hormone production [15]. Perchlorate is an environmental toxicant found in food and drinking water that interferes with iodide transport through the NIS. By blocking active transport and substituting for iodide, perchlorate can both reduce thyroidal iodine stores and accumulate in the gland [15,16]. These actions lead to a decrease in TH synthesis and reductions in serum TH concentrations, which have been well documented in rodent models [17,18,19,20,21,22]. Although pregnant women and children with iodine deficiency represent the most vulnerable populations of concern for perchlorate exposure [23], there is surprisingly little experimental data examining the potential interaction between ID and perchlorate exposure on brain development.

Using a rodent pregnancy model, we previously reported dose-dependent disruptions of perchlorate exposure on the thyroid axis and developing brain [20,21,22,24]. We similarly reported the impact of varying degrees of dietary iodine insufficiency in the brain using the same rodent model [25,26,27]. Dose-dependent reductions in serum thyroxine (T4) in the pregnant dam were accompanied by decreases in fetal serum and brain hormones with both manipulations [21,26]. However, except for electrophysiological measures of hippocampal synaptic transmission [20], only relatively minor effects on brain and behavior were evident in the offspring of ID or perchlorate-treated dams [17,20,21,28,29,30]. We recently reported a robust increase in the size and prevalence of a structural malformation, a periventricular heterotopia (PVH), in the neonatal rat brain when maternal dietary ID was paired with perchlorate conditions that, by themselves, failed to reduce THs in offspring and resulted in only minor or no effects on brain structure [24]. A PVH is a cluster of neurons that have failed to migrate appropriately. Their presence is indicative of a disruption of TH signaling in the brain during a critical window of development. We hypothesized that other measures of altered brain structure and function may also accompany exposure to perchlorate when delivered under conditions of ID. Here, we examine TH profiles and the expression of TH-responsive genes in the thyroid gland and brain of the neonate in this same cohort of animals. We extend our observations to include an evaluation of inhibitory neurons and two measures of neurobehavior in adult offspring.

## 2. Materials and Methods

### 2.1. Subjects

Long–Evans (LE) female (n = 96) rats 45–48 days of age were obtained from Charles River (Raleigh, NC, USA) and pair-housed in standard plastic hanging cages, with pine shavings as bedding. Immediately upon arrival, all were given free access to deionized drinking water in an approved animal facility. Counterbalanced by weight, half of the animals were provided with a control iodine-replete diet (D10001, Research Diets, nominally containing 225 ng iodine/gm of chow) and the other half were given an iodine-deficient diet (25 ng iodine/gm of chow) (Research Diets, Newark, NJ, USA). Based on previous work, these diets led to a mean daily iodine intake of 4.0 vs. 0.47 µg I/day in the two groups prior to breeding [25,26,27]. After a minimum of 4 weeks on these diets, pairs of females in proestrus were placed with one LE breeder male overnight and vaginal smears were assessed for the presence of sperm the following day. Females who were not sperm-positive remained on designated chow and were rebred at the next most convenient proestrus opportunity. Sperm-positive females from each diet group were either maintained on the control (0 ppm) or switched to ammonium perchlorate (300 ppm ClO_4_ dissolved in deionized water, CAS No. 7790-98-9, purity 99.5%, Sigma, St. Louis, MO, USA) containing drinking water on gestational day (GD) 6. All dams were maintained on these dietary and drinking water regimens until the weaning of the pups on postnatal day (PN) 21. This created a simple 2 × 2 design with four treatment conditions: Control–Control (Con-Con), Iodine Deficient–Control (ID-Con), Control–Perchlorate (Con-ClO_4_), and Iodine Deficient–Perchlorate (ID-ClO_4_), with the designation before the hyphen representing the iodine nutritional manipulation initiated prior to breeding and the designation after the hyphen representing the presence or absence of perchlorate in the drinking water beginning on GD6. The dose of perchlorate and dietary iodine deficiency level were based on previous work from our laboratory, where an extensive dose–response analysis was conducted for perchlorate [20,21,22] and dietary iodine [25,26,27]. Some findings from this study were previously reported by Gilbert et al. [24].

Prior to breeding, blood was collected from the tail vein of a subset of animals from both dietary groups 4 weeks after the initiation of treatment. Additional samples were collected at 8 weeks from females prior to additional breeding attempts. A final blood sample was collected from females who had remained in the study but were not successfully bred after 15 weeks. Both control and ID chow were equally represented in this final group. The final number of successful pregnancies was 10–15 dams in each treatment condition. Dam body weights were monitored frequently throughout pregnancy and litters were weighed by sex at several intervals after birth until weaning. All experiments were conducted with prior approval from the United States Environmental Protection Agency’s Institutional Animal Care and Usage Committee (IACUC) and carried out in an Association for Assessment and Accreditation of Laboratory Animal Care (AAALAC)-approved facility. Animal rooms were maintained with a 12:12 light/dark schedule and animals were permitted free access to food and drinking water throughout the study.

### 2.2. Tissue Collection

Dam blood was collected from the tail vein on GD16 and GD20 as previously described [29]. Trunk blood was collected from pups on PN0, PN2, PN6, and PN14 and from dams on PN21, when pups were weaned. Animals were humanely euthanized by decaptitation without anesthesia. Depending on the litter size, multiple pups were collected on PN0 and litters were culled to 10 on PN2. As previous studies did not report sex differences in perchlorate-treated animals at PN14 or PN21 [20], serum was pooled for all animals collected from the same litter at each age, regardless of sex. Samples were allowed to clot on ice and centrifuged and serum was collected and stored at −80 °C until analysis. Sample collection was completed between the hours of 8:00 am and 12:00 pm, and each dose group was consecutively sampled to control for diurnal fluctuations in thyroid hormones.

Thyroid glands were collected from pups at the time of sacrifice on PN0, PN2, PN6, and PN14 and from dams on PN21 and flash frozen for gene expression, hormone, and perchlorate analyses. Thyroid glands were pooled across pups within a litter of the same age group. Gland weights were taken from PN14 pups and dams. On PN14, an oblique section of the frontal/parietal cortex and hippocampus were collected from one male pup per litter, rinsed in cold phosphate-buffered saline, and saved in RNAlater™ (Invitrogen, Carlsbad, CA, USA), according to the manufacturer’s recommendation. All samples were subsequently stored at −80 °C for transcriptomic analysis. The brains of one male and one female from each litter were collected on PN14 for immunohistochemistry, as described below.

### 2.3. Perchlorate Analysis in Serum and Milk

Perchlorate was extracted from the serum of dams and pups on PN0, PN2, PN6, and PN14 and milk bands on PN2, according to methods modified from Oldi and Kannan [30], as reported in Gilbert et al. [22]. Briefly, samples were thawed to room temperature. Milkbands were homogenized to 4× the weight with deionized 1% acetic acid in water. A 50 µL aliquot of serum or milk homogenate was transferred to a 1.5 mL Eppendorf centrifuge tube and diluted with deionized water. The internal standard (Cl^18^O_4_) (10 µL of 25 µg/mL) was added to 0.5 mL of the diluent in a Vivaspin 500 centrifugal concentrator (30,000 molecular cutoff) and vortexed to mix. The samples were filtered by centrifugation at 3000× *g* for 2 min. An aliquot of the filtrate (50 µL) was diluted with deionized water (200 µL) in an autosampler vial for analysis and analyzed by Liquid Chromatography Mass Spectrometry (LC/MS/MS) using an AB Sciex (Framingham, MA, USA) Exion AC UHPLC-Qtrap 6500^+^ Linear Ion Trap LC/MS/MS system.

### 2.4. Quantification of THs in Serum

#### 2.4.1. T3 and T4 in Serum

Total T3 and T4 were analyzed in serum using the same LC/MS/MS system described above for perchlorate analysis and according to previously described methods [29,31]. Solvent-based calibration standards were used for quantitation over a range of 10–10,000 pg/mL in a 100 µL standard curve volume. Two ion transitions were monitored for each target analyte, qualitatively identified based on retention time relative to the internal standard and calibration standard, and the ratio of the peak areas of the monitored ion transitions. The lower limit of quantitation (LLOQ) for each analyte was set to the concentration of the lowest calibration standard that gave an acceptable ion ratio, with an acceptable recovery of ±30% of the spike amount. Each sample batch consisted of a method blank, a laboratory control sample (blank spike), and a continuing calibration verification sample prepared in a solvent. The lower limit of quantification (LLOQ) for both T4 and T3 was 0.1 ng/mL.

#### 2.4.2. Thyroid-Stimulating Hormone

The thyroid-stimulating hormone (TSH) was measured by radioimmunoassay (RIA) using a standard double-antibody assay, as previously described [19,22]. The TSH was radiolabeled with ^125^I (Perkin Elmer, Boston, MA, USA) by a modification of the chloramine-T method of Greenwood et al. [32] described in Goldman et al. [33]. A rat TSH standard curve of 0.0195 ng/mL to 20.0 ng/mL was prepared with 100 μL of standard reference concentrations. All serum samples were run in duplicate in the RIA assay. For dams and older neonates, a volume of 50 µL serum was analyzed, whereas the serum volumes for younger neonates were reduced to 25 µL due to a limited sample volume. The limit of detection was 1.10 ng/mL and the intra-assay CV for the assay was 3.52%.

### 2.5. Gene Expression in Thyroid Gland and Brain by Quantitative Real-Time PCR

Gene expression was assessed in the thyroid glands of dams and pups at various ages and the cortex, hippocampus, and cerebellum of pups on PN14, according to standard procedures described by O’Shaughnessy et al. [34]. Total RNA was extracted via TRIzol^®^ (Invitrogen), treated with DNase I (Promega, M6101, Madison, WI, USA), and quantified using the Ribogreen Quantitation Kit (ThermoFisher, R11490, Norristown, PA, USA). RNA samples were then reverse-transcribed using the ABI cDNA Archive Kit (ThermoFisher, 4322171) with random primers. cDNA (25 ng) was amplified using the TaqMan™ Gene Expression Assays (Thermo-Fisher) and Gene Expression Master Mix (ThermoFisher, 4369016). Quantitative real-time PCR (q-PCR) was performed on a 7600HT Fast Real-Time PCR System (Applied Biosystems, Foster City, CA, USA). The thermocycler program was 95 °C for 5 min, followed by 40 cycles of 15 s at 95 °C and then 1 min at 60 °C. Each sample was run in duplicate, and the data were analyzed using the 2^−ΔΔCT^ method [35]. All samples were amplified with β-2microglobulin (*β2m*) or beta actin (*β-actin*) to select a housekeeping gene that was not altered by treatment, as verified by one-way ANOVAs (*p* = 0.10 or higher). Once the most stable gene for each tissue type was identified, that gene was selected as the reference gene for that tissue type and run again on each plate with the genes of interest. For the thyroid gland, cortex, and cerebellum, *β2m* served as the reference gene. For the hippocampus, *β-actin* was chosen for this purpose. Target and reference genes assayed in different tissues are summarized in Supplementary Table S1.

### 2.6. Immunohistochemistry for Parvalbumin

#### 2.6.1. Tissue Collection and Immunohistochemistry Procedures

Brains collected on PN14 from one male and one female pup per litter were immersion-fixed for 5 days in 4% paraformaldehyde and subsequently transferred to a cryoprotectant solution until sectioning on a vibratome (60 µm). Sections from the posterior regions of these same brains were sequestered to examine the presence of a heterotopia, the results of which are reported by Gilbert et al. [24]. The remaining anterior sections containing the somatosensory and cingulate regions of the anterior cortex and the auditory cortex in more posterior cortical regions were prepared for immunohistochemistry with either parvalbumin (Pvalb) or GAD-67. Free-floating immunohistochemistry was conducted according to standard procedures as previously described [31,36,37,38]. In general, sections were incubated overnight at 4 °C in a primary antibody (Pvalb Swant, 1:2000 or GAD-67 Abcam, 1:1000 dilution), followed by a biotinylated secondary antibody (1:400) in conjunction with avidin–biotin amplification (Vectastain Elite ABC, Vector, Burlingame, CA, USA), using diaminobenzidine tetrahydrochloride (DAB) as the chromogen. To improve staining for GAD-67, antigen retrieval procedures were conducted by placing sections in 14 mL falcon tubes filled with a 10 mM citrate buffer at pH 6 without Tween-20. Tubes were placed in a heated (90 °C), shaking water bath for 30 min, followed by a Tris-buffered saline (TBS) wash and immunohistochemistry thereafter. TBS replaced TBS-X in all immunohistochemistry buffers for GAD-67 to reduce cell rupturing and optimize stain visualization. Upon the completion of staining, sections were mounted on gelatin-coated glass slides, dried, and Pvalb sections were lightly counterstained with cresyl violet prior to coverslipping.

#### 2.6.2. Imaging and Section Selection for Cell Counting

Sections were imaged using an Aperio AT2 slide scanner (Leica Biosystems, Buffalo Grove, IL, USA). Automated counting of Pvalb+ neurons in three cortical regions was conducted using Aperio ImageScope software (Leica Biosystems, Buffalo Grove, IL, USA). To quantify Pvalb+ neurons in the cortex, a rectangle sufficiently large to encase the cingulate cortex (Cg) (6.6 mm high × 0.3 mm wide) or a portion of the somatosensory cortex (SS) (0.3 mm high × 5.4 mm wide) was positioned on a series of anterior sections of both hemispheres. Similarly, a rectangle encompassing the auditory cortex (Aux) (4.2 mm high × 0.4 mm wide) was placed on posterior sections. The anterior sections of cortex examined spanned the region just anterior to the septum (~1.6 mm anterior to bregma) to the emergence of the dorsal hippocampus (~1.4 mm posterior to bregma), roughly corresponding to Plates 12 to 24, (*Atlas of Paxinos and Watson*, *2nd Edition*) [39]. The posterior sections ranged from Plates 40 to 43 (~5.6–6.3 posterior to bregma) of the atlas. Mean cell counts across all sections in both hemispheres were calculated for each animal in each region. Cortical cell counts were conducted on consecutive sections (median = 7–12 sections/animal) from 8–12 male rats/treatment group. For females, every 3rd section was selected for staining (median = 7–8 sections/animal) and subjected to counting (7–10 female rats/treatment group). Left and right hemispheres were compared and mean counts across all sections were calculated for male and female rats in each region and subjected to statistical analysis.

#### 2.6.3. Quantification of Parvalbumin-Expressing Neurons

To optimize and validate the automated counting procedures, direct comparisons were made between automated counts and those identified by direct visual inspection. The software parameters were optimized to reliably quantify only Pvalb^+^ neurons. To verify automated procedures, a subset of samples manually counted by two independent observers revealed high inter-rater reliability (CV < 3.6%), and similar counts were returned from automated vs. manual counts (CV < 4.4%).

### 2.7. Neurobehavioral Assessments in Adult Offspring

#### 2.7.1. Trace Fear Conditioning

Adult offspring were assessed for learning and memory using a distract trace fear conditioning paradigm as previously described [40,41,42]. Females were assessed one week (age range PN56–78) and males two weeks (PN75–92) later. On the first day of fear conditioning, animals were placed in a test chamber (Habitest, Coulbourn Instruments, Allentown, PA, USA) equipped with a small animal shock generator (H13–16, Coulbourn Instruments). The chamber was sprayed with Windex™ cleaner to provide a distinctive olfactory cue during training and context testing. The training procedure consisted of a 2 min baseline period followed by the onset of a 15 s compound light/tone conditioned stimulus (CS). The unconditioned stimulus (US) was a scrambled foot shock (1 mA, 2 s duration) delivered through a metal grid floor. Two CS-US pairings were delivered, two minutes apart. CS offset and US onset were separated by a 30 s trace interval. A non-contingent distractor stimulus (a dim light on the wall opposite the cue light flashing for 3 s) was randomly presented throughout the training session. This was introduced to increase the task difficulty and engage attentional brain circuitry in addition to the amygdala (CS-US association) and hippocampus (context and trace element). Conditioning to context was assessed the following day by returning animals to the same test chamber for 5 min in the presence of the olfactory Windex™ cue but with the absence of explicit CS, UCS, or distractor stimuli. Approximately one hour later, conditioning to cue was assessed in a room distinct from training and context testing in a similarly sized test box decorated with striped walls and a smooth floor. The context was further differentiated from the training environment by leaving the enclosure doors open with room lights on, and the distinct olfactory cue present during training was absent. A protocol similar to that used during training was employed with only a single CS presentation after the 2-min baseline period; no shock US was delivered and no distractor stimuli were presented. Activity was recorded for three minutes after CS presentation.

Activity in each phase of testing was quantified using an infrared motion detector (Colbourne Instruments) mounted on the ceiling of each test chamber. Reduced activity was taken as the measure of fear-induced learning, with greater suppression indicating better learning. Activity counts were recorded in 15-s bins for training, context, and cue testing. Conditioning to context was measured as activity counts during the first 2 min of testing on Day 2, compared to the number of counts recorded during the 2 min before delivery of the CS on Day 1. Conditioning to cue was taken as the mean number of activity counts recorded in the novel test chamber on Day 2 in the 30 s trace period after CS presentation compared to the mean counts/30 s during the 2-min prestimulus baseline period activity before CS delivery.

#### 2.7.2. Prepulse Inhibition (PPI) of Acoustic Startle Response (ASR)

The PPI of ASR represents a basic inhibitory process regulating sensory inputs, with the magnitude of the PPI serving as a measure of the behavioral salience of perceptually relevant environmental stimuli [43]. ASR and PPI testing were conducted a minimum of one week following the completion of fear conditioning. Basal levels of the ASR, habituation of ASR, and inhibition of ASR by administration of a prepulse noise (PPI) were measured using the TSE Startle Response SystemTM (TSE Inc., Chesterfield, MO, USA). Animals were placed into one of four TSE Systems test boxes housed within separate sound-dampening acoustical chambers (IAC No. AC-361) located in a sound-attenuated test room. Loudspeaker calibrations were conducted following the manufacturer’s instruction (Version 3.09, TSE Startle Response System, TSE Inc., Chesterfield, MO, USA) and all noise levels were measured using a ¼ inch pressure field microphone, Model 4938, attached to a pre-amplifier, Model 2639, and a measuring amplifier, Model 2636-D (all equipment Bruel and Kjaer, Virum, Denmark). Prior to the test, animals were gently handled for a minimum of 3 days and acclimated to the acoustic startle chambers for 10 min the day prior to testing. On the day of testing, after a 5 min baseline period, 30 ASR trials were delivered against a 65 dB background noise at a fixed intertrial interval (ITI) of 20 s. The startle stimulus was a 40 ms burst of white noise at 115 dB, SPL. A highly sensitive transducer (weight sensor) integrated into the measuring platform recorded dynamic changes from the animal’s reactions during this acquisition period (TSE startle response system software, version 3.09). The ASR was measured as the maximum amplitude in grams (MaxG) occurring within 100 ms of stimulus delivery. ASR habituation data were averaged across each 5-trial block for each animal, yielding 6 blocks of ASR MaxG data for the analysis of baseline ASR amplitudes and ASR habituation.

Prepulse inhibition (PPI) of the ASR was assessed immediately following ASR habituation. Animals received a total of 60 trials using a sensorimotor gating paradigm [44] incorporating both prepulse and no-prepulse trials. These trials encompassed 6 trial types: blank trial where no stimulus was delivered on top of the 65 dB background noise, startle stimulus only with no prepulse (PP), and 4 different 20 ms white noise PP trials consisting of stimuli of 3, 6, 10, and 18 dB above the 65 dB background noise. The onset of each PP was fixed, preceding the onset of the startle stimulus by 60 ms (interstimulus interval or ISI). Each trial type was delivered 10 times throughout the session in a pseudo-random order, programmed to occur once within each block of 6 trials to ensure equal distribution across the PPI assessment. Each PPI trial was preceded by a variable ITI between 20 and 30 s in duration. Each of the 6 PPI trial types was averaged across the 10 trials to yield one MaxG measurement per PPI trial (Blank, AS stimulus alone, 3 dB PP, 6 dB PP, 10 dB PP, and 18 dB PP). PPI was defined as the percent decrease in the MaxG for each of the prepulse trials (PP) relative to the MaxG in response to AS stimulus alone: PPI = (MaxG of PPdB/MaxG of ASR) × 100.

### 2.8. Statistical Analysis

All data were expressed as the mean ± standard error mean (SEM). For measures taken across time, repeated-measure analysis of variance (ANOVA) was performed to determine the main effects of treatment, sex, age, and interactions where appropriate (SAS Version 9.4, Cary, NC, USA). Step-down one-way ANOVAs were conducted when significant effects were observed in the overall ANOVA. When significant treatment effects were detected in the step-down ANOVAs (α = 0.05), pairwise comparisons were conducted using Dunnett’s adjustment for multiple comparisons. For gene expression analysis, a minimum fold-change was set at 1.5 for gland and 1.25 for brain, with a 5% false discovery rate. To further control for experiment-wise error in these data, α was further reduced to 0.02 by dividing 0.05 by the square root of the number of targets examined. The ANOVA of the mean number of Pvalb^+^ cells for male and female rats was evaluated for effects of treatment, region, and sex. Step-down ANOVAs were conducted for each brain region. With the exception of behavioral assessments as described below, the litter was the unit of analysis for all measures.

Behavioral tests were conducted on 1–2 male and female offspring from any given litter. Dependent variables were examined for normality using the Shapiro–Wilk test and, if the data were judged to be non-normal, the dependent measure was transformed to achieve normality. In trace fear conditioning, both context and cue learning assessed the difference between baseline and testing activity counts as the dependent variable in mixed linear models fitted in the SAS MIXED procedure, as described in Steel and Torrie [45]. For context learning, the mean 30 s count in the test box during baseline on Day 1 prior to CS/US delivery was subtracted from the mean 30 s count calculated over the first 2 min in the test box on Day 2. For cue testing, the dependent variable was mean counts during baseline in the novel box on the same day as cue testing subtracted from the mean counts during the trace period following CS delivery.

All acoustic startle data were transformed to a log_10_ scale and data were analyzed in separate mixed-model ANOVAs with treatment as a between litter factor and block (ASR habituation) or dB (prepulse inhibition) as repeated measures. Various covariance structures were examined as appropriate for the type of repeated factor and the best-fitting model was selected based on AICC values [46].

For all behavioral data, where more than one male or female pup was tested from any given litter, the random effect of litter was used to account for variation between litters and modeled using the (default) variance components covariance structure [47,48]. Differences in the least-squares means were used to contrast treatment groups vs. controls, applying Bonferroni adjustment for multiplicity.

## 3. Results

### 3.1. Body Weight

**Figure 1:**
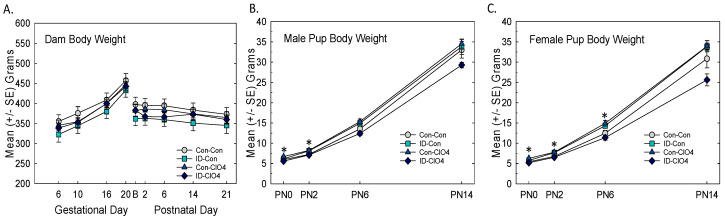
Body weights. (**A**) Mean (+/−SE) body weight did not differ across treatment conditions in pregnant and lactating dams (n = 10–13 dams/treatment). (**B**) Treatment-related body weight increases were seen in both male (**B**) and female (**C**) pups from the Con-ClO_4_ relative to the Con-Con group at these young ages. * Dunnett’s *t*-test, significantly different from Con-Con using α = 0.05. B-Birth.

Dam body weight increased over gestation as expected and remained stable during the postnatal period (Figure 1A). There were no differences among treatment groups [treatment: F_(3,43)_ = 0.69, *p* = 0.56; treatment and age interaction: F_(8,24)_ = 0.35, *p* = 0.37]. No statistically significant reductions in body weight were detected in male or female offspring at any age tested (Figure 1B,C). Slight increases in body weight were detected at very young ages in the Con-ClO_4_ group relative to Con-Con in both males [treatment F_(3,35)_ = 3.45, *p* = 0.0242] and females [treatment F_(3,46)_ = 8.27, *p* = 0.0002]. A significant treatment and age interaction was seen in females [F_(9,138)_ = 4.8, *p* = 0.0034] but not in males [F_(9,135)_ = 1.47, *p* = 0.16].

### 3.2. Serum Thyroid Hormones: Pre-Breeding, Gestation, Lactation

**Figure 2:**
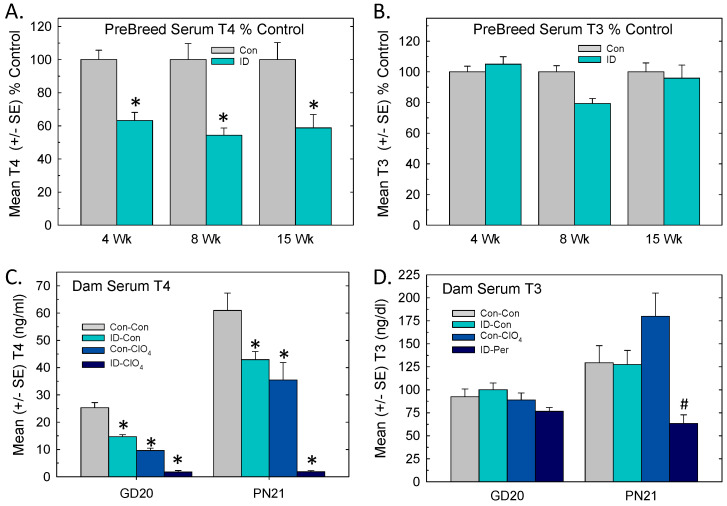
Serum hormones before and after pregnancy. (**A**) Mean (+/−SE) serum T4 and (**B**) T3 in control and ID female rats prior to breeding. (**C**) Dam serum T4 was reduced in all treatment groups relative to Con-Con in late gestation and at weaning of pups on PN21. (**D**) Dam T3 did not differ among groups in late gestation but significant differences in T3 were present on PN21. * Dunnett’s *t*-test, significantly different from Con-Con using α = 0.05; # Dunnett’s *t*-test, significantly different from Con-ClO_4_ using α = 0.05. Sample size range 8–16/treatment group at pre-breeding and 12–15/treatment group at GD20 and PN21.

Significant drops in serum T4 were observed in female rats maintained on ID chow prior to breeding (Figure 2A). These reductions were evident by 4 weeks on the diet [F_(1,61)_ = 48.6, *p* < 0.0001] and remained stable over the next 11 weeks. Serum T3 in ID rats did not differ from Con at any timepoint [F_(1,61)_ = 0.76, *p* = 0.38] (Figure 2B).

During pregnancy and lactation, dam serum T4 was reduced in all treatment groups relative to Con-Con (Figure 2C). ID-ClO_4_ dams exhibited the greatest decreases relative to Con-Con dams at both time points. An overall ANOVA supported significant main effects of treatment [F_(3,96)_ = 7.52, *p* < 0.0001], time [F_(1,96)_ = 14.6, *p* < 0.0001], and treatment and time interaction [F_(3,96)_ = 5.41, *p* = 0.0025]. Step-down ANOVAs by time supported main effects of treatment at both ages (*p* < 0.0001) and Dunnett’s *t*-test supported significant reductions in all treatments relative to Con-Con.

Serum T3 did not differ among groups in dams at late gestation (F_(3,50)_ = 1.9, *p* = 0.13]. Significant differences in dam T3 were present on PN21 [F_(3,46)_ = 8.81, *p* = 0.0007]. Dunnett’s *t*-test confirmed that serum T3 was lower in the ID-ClO_4_ group relative to the Con-ClO_4_ group, but ID-CLO_4_ did not differ from Con-Con (Figure 2D).

### 3.3. Serum Thyroid Hormones and TSH in Offspring

**Figure 3:**
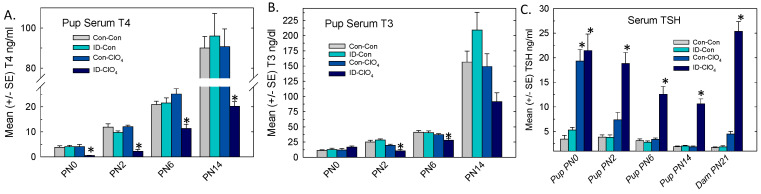
Pup serum hormones. Mean (+/−SE) serum T4 (**A**) and T3 (**B**) in pups and TSH (**C**) in pups and dams. Reductions in serum T4 were present at all ages and were limited to the ID-ClO_4_ relative to the Con-Con group. Reductions in serum T3 were also evident in this treatment group on PN2 and PN6 but were not significant on PN14. (**C**) Pup serum TSH was increased in the ID-CLO_4_ group relative to the Con-Con group at all ages tested and in dams on PN21. On PN0, TSH was significantly elevated above that of the Con-Con group in both perchlorate-exposed groups. * Dunnett’s *t*-test, significantly different from Con-Con using α = 0.05. Sample size for T3 and T4 ranged from 7 to 14 litters on PN0 and 10 to 15 at older ages. Sample size for TSH was 5–12 litters on PN0 and 9–15 at older ages and in dams.

In pups, serum T4 was decreased at all ages (Figure 3A), but in a pattern that was distinct from dams. The results of an overall ANOVA revealed significant main effects of treatment [F_(3,160)_ = 37.7, *p* < 0.0001], age [F_(3,160)_ = 2.69, *p* < 0.0001], and treatment and age interaction [F_(9,160)_ = 16.49, *p* < 0.0001]. Significant step-down ANOVAs at each age (*p* = 0.0003 or less) supported reductions in serum T4 at all ages that were limited to the ID-ClO_4_ relative to the Con-Con group.

The results of the analysis of pup serum T3 are summarized in Figure 3B. An overall ANOVA supported significant main effects of treatment [F_(3,160)_ = 4.62, *p* = 0.004], age [F_(3,160)_ = 144.9, *p* < 0.0001], and treatment and age interaction [F_(9,160)_ = 4.24, *p* < 0.0001]. Step-down ANOVAs at each age yielded significant reductions in serum T3 at PN2 [F_(3,46)_ = 12.15, *p* < 0.0001] and PN6 [F_(3,38)_ = 4.70, *p* = 0.0069]. Mean contrast tests revealed that at the younger ages, reductions in serum T3 were limited to the ID-ClO_4_ group. Although a significant effect of treatment was also seen for pup serum T3 on PN14 [F_(3,46)_ = 4.90, *p* = 0.0054], multiple comparison tests did not distinguish among groups.

Pup TSH was increased (Figure 3C), with the overall ANOVA revealing significant effects of treatment [F_(3,136)_ = 96.18, *p* < 0.0001], age [F_(3,136)_ = 39.18, *p* < 0.0001], and treatment and age interaction [F_(9,136)_ = 9.29, *p* < 0.0001]. Step-down ANOVAs at each age supported significant TSH increases in pup serum at PN0 [F_(3,27)_ = 28.28, *p* < 0.0001], PN2 [F_(3,35)_ = 26.14, *p* < 0.0001], P6 [F_(3,36)_ = 36.36, *p* < 0.0001], and PN14 [F_(3,38)_ = 68.13, *p* < 0.0001]. Serum TSH was also increased in the dams when tested at the time of weaning of pups on PN21 [F_(3,49)_ = 140.69, *p* < 0.0001]. Mean contrast tests showed that TSH increases were limited to the ID-ClO_4_ group in dams and pups at all ages, with an additional significant increase present in the group exposed to perchlorate alone (Con-ClO_4_) on PN0. Exposure to ID alone did not affect TSH at any time point.

### 3.4. Thyroid Gland: Weight and Gene Expression

**Figure 4:**
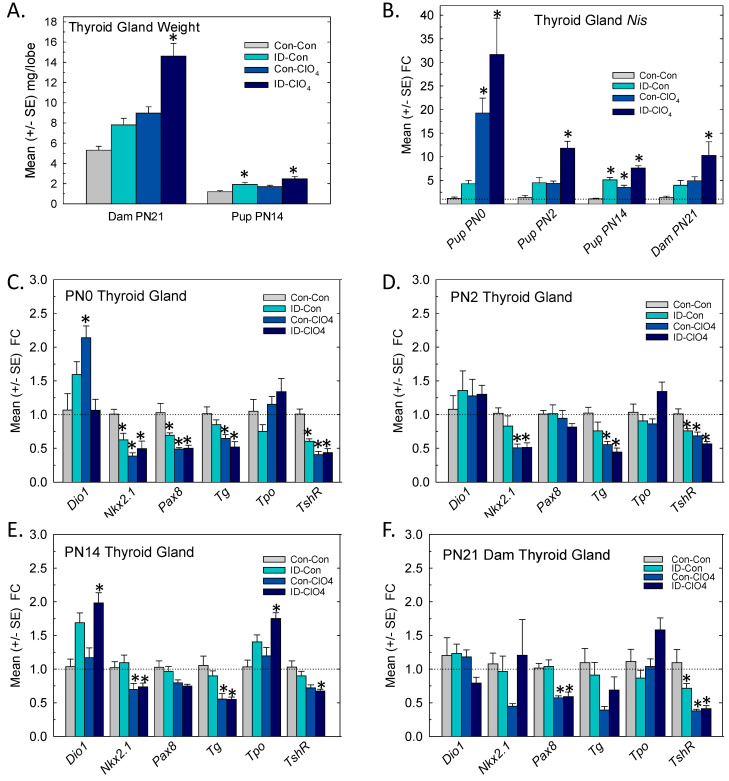
Thyroid glands. (**A**) Mean (+/−SE) thyroid gland weight increased significantly in dams and pups. (**B**) *Nis* was upregulated in the thyroid glands of pups at all ages, with the most consistent increases across age seen in the ID-ClO_4_ group. (**C**–**E**) Thyroid gland transcripts were differentially affected by treatment and age, with a prominent pattern of downregulation in both perchlorate-exposed groups. (**F**) Gene expression changes in dams on PN21 were limited to the downregulation of Pax8 in both perchlorate-exposed groups and of *TshR* in all treatment groups relative to the Con-Con group. * Dunnett’s *t*-test, significantly different from Con-Con using α = 0. 05. Dotted line indicates FC = 1. Sample sizes ranged from 4 to 7/treatment on PN0 and 6 to 8 for older-aged pups and dams.

Significant increases in thyroid gland weight were seen in dams and limited to the ID-ClO_4_ group [F_(3,46)_ = 15.04, *p* < 0.0001] (Figure 4A, left). Small but significant increases in PN14 pup thyroid gland weight [F_(3,46)_ = 9.21, *p* < 0.0001] were present in the ID-Con and ID-ClO_4_ groups (Figure 4A, right).

The differential expression of seven genes critical for TH synthesis was assessed in the thyroid glands of pups and dams, and the results are summarized in Figure 4B–F. In the glands of newborn pups, a dramatic upregulation of *Nis* expression was prominent, increases of ~20-fold were present in Con-ClO_4_ group, and increases exceeding 30-fold were present in animals from the ID-ClO_4_ group (Figure 4B). As pups aged, the upregulation of *Nis* subsided but remained elevated in the ID-ClO_4_ throughout the neonatal period. The results of an overall ANOVA supported these observations of the increased differential expression of *Nis* in the pup thyroid glands, yielding significant main effects of treatment [F_(3,67)_ = 22.4, *p* < 0.0001], age [F_(2,67)_ = 24.4, *p* < 0.0001], and treatment and age interaction [F_(6,67)_ = 8.1, *p* < 0.0001]. *Nis* was also upregulated in dam thyroid glands when assessed on PN21 [F_(3,28)_ = 5.57, *p* = 0.004] where significant increases, similar in magnitude to those observed in pups on PN14 (~10-fold), were limited to the ID-ClO_4_ group.

The expressions of the other six transcripts examined in the thyroid glands of pups are summarized in Figure 4C–E. Relative to *Nis*, the magnitude of change in these transcripts was comparatively small and the predominant pattern was downregulation. One exception was Deiodinase 1 (*Dio1*), the transcript for the primary metabolizing enzyme in the thyroid gland, where expression was increased, but this upregulation was inconsistent across ages and treatment conditions. Differential expression of thyroglobulin (*Tg*), the transcript encoding the primary protein substrate for TH biosynthesis, was reduced by perchlorate at all ages tested. In the regulatory negative feedback loop, activation of the TSH receptor (TshR) was induced to counter declines in serum T4 by upregulating *Nis*, *Tg*, and thyroid peroxidase (*Tpo*) genes. TshR activation also modulated the expression and activity of the transcription factors *Nkx2.1* and *Pax8* [49].

In the present study, increases in *Nis* expression on PN0 were accompanied by the downregulation of *TshR*, *Nkx2.1*, and *Pax8* in all treatment groups. Reductions in the relative expression of *Tshr*, *Nkx2.1*, and *Tg* persisted to PN14 but were largely limited to the two groups exposed to perchlorate (Con-ClO_4_ and ID-ClO_4_). The expression of *Tpo* was increased in dam and PN14 pup glands, although not at the younger ages. The downregulation of *Tshr* and *Pax8* was also present in perchlorate-exposed dams (Figure 4F). The results of an overall ANOVA for treatment, age, and treatment and age interactions and step-down ANOVAs for each gene at each age are summarized in Supplementary Table S2. Significant differences based on least-squares means adjusted for multiple comparisons in genes reaching the FC cutoff of >1.5 and more stringent α = 0.02 criteria are indicated in Figure 4.

### 3.5. Perchlorate in Serum and Milk

**Figure 5:**
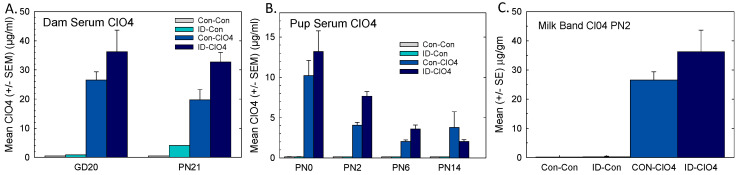
Dam and pup perchlorate in serum and milk. (**A**) Mean (±SEM) serum perchlorate in dams during late gestation and at the end of lactation and (**B**) pups over the first two weeks of life. (**C**) Perchlorate was present in the milk bands of pups on PN2 and at higher concentrations than in the serum at the same age. Sample sizes per treatment group were 6 for serum and 5–7 for milk band.

Perchlorate was measured in the serum of pregnant and lactating dams. Negligible levels were present in samples from the Con-Con and ID-Con groups, and higher concentrations were achieved in the ID-ClO_4_ group relative to exposure to ClO_4_ alone (Figure 5A).

Perchlorate was also present in pup serum at all ages but at much lower levels than that seen in dams (Figure 5B). Pup serum ClO_4_ was at its highest on PN0 and declined over the next 2 weeks, despite continued maternal exposure. Perchlorate was also measured in milk bands collected from pups on PN2, and approximated levels observed in dam serum were surprisingly 2 times higher than perchlorate concentrations in pup serum (Figure 5C). Higher serum and milk band perchlorate levels in the co-exposed group suggested greater accumulation when perchlorate was administered under conditions of ID. This may have been because Nis transporters are not only present in the thyroid gland but also in the intestine and mammary gland [50]. In the present study, a greater upregulation of *Nis* occurred in the thyroid gland when perchlorate exposure was combined with ID. Perchlorate not only blocks iodide passage through the Nis but can substitute for iodide to enter tissues directly via the Nis [16]. It is possible that the upregulation that we observed in the thyroid gland of *Nis* also occurred in these other Nis-expressing tissues, contributing to a greater perchlorate accumulation in serum and milk in the co-exposed group.

### 3.6. TH Action in the Brain

**Figure 6:**
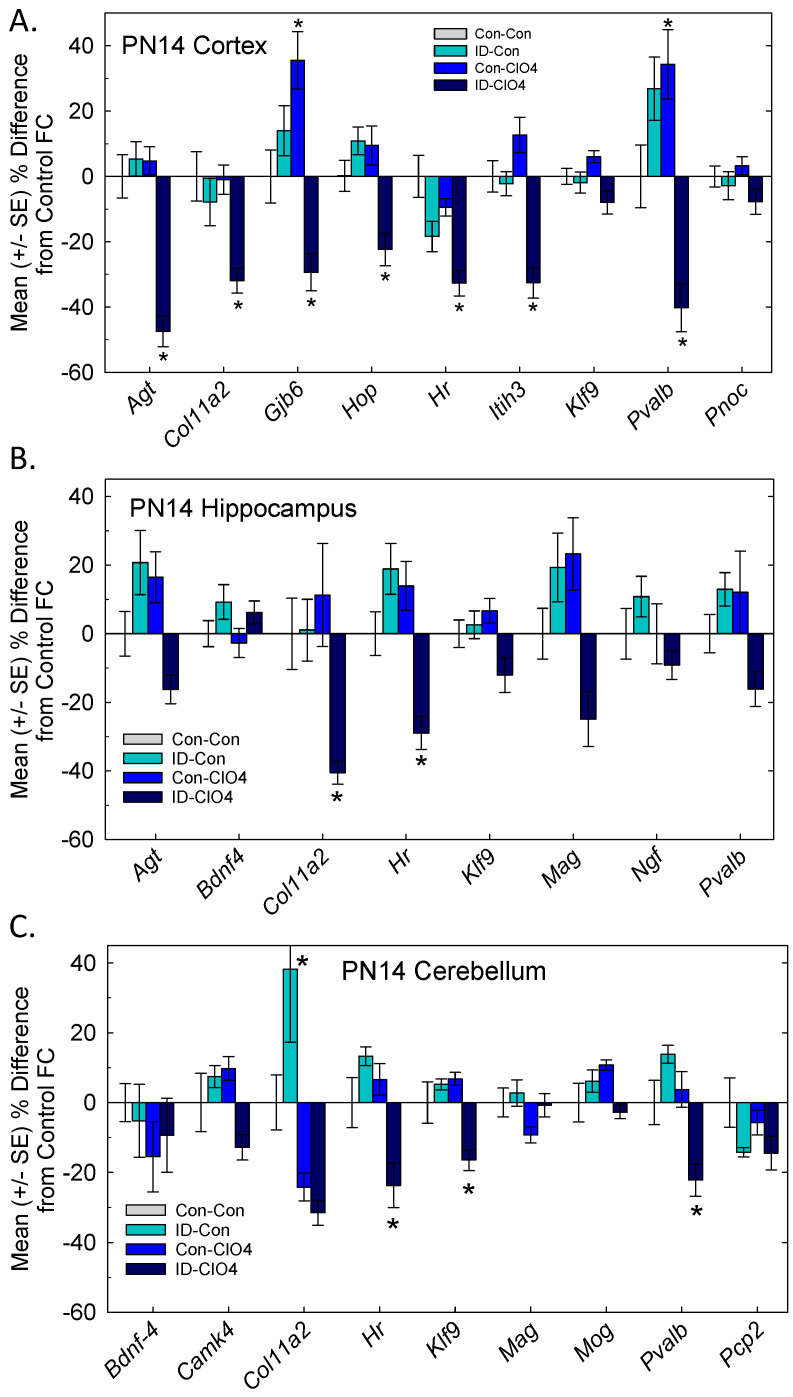
Thyroid hormone action in brain. Mean (±SE) relative expression of TH^−^ responsive genes in the brains of rat pups on PN14. A predominant pattern of downregulation was evident, largely limited to the ID-ClO_4_ treatment condition. Relative expression was significantly lower in several transcripts in the cortex (**A**), hippocampus (**B**), and cerebellum (**C**). * Dunnett’s *t*-test, significantly different from Con-Con using α = 0.05. Sample size varied from n = 7–12/treatment condition for cortex and hippocampus and n = 4–6 for cerebellum.

A set of TH-responsive genes previously identified in rat pups exposed to propylthiouracil (PTU) [36] were examined in the cortex, hippocampus, and cerebellum of the PN14 pups (Figure 6). More genes were altered in the cortex than the other two brain regions and reductions in relative gene expression were largely limited to the ID-ClO_4_ group. *Hr*, a TH-specific transcription factor, was downregulated in all three regions examined. *Klf9*, the other TH-dependent transcription factor, was downregulated in the cerebellum. *Col11a2*, an extracellular matrix adhesion molecule, was downregulated in the cortex and hippocampus, and *Pvalb*, a calcium-binding protein present in a subset of inhibitory neurons, was reduced in the cortex and cerebellum. Additional transcripts in the cortex were found to have significant downregulation, including *Agt*, *Gjb6*, *Hop*, and *Itih6*. Significant treatment effects were detected for *Klf9* in the cortex and *Pvalb* and the myelin protein *Mag* in the hippocampus, but no group differences were found. Detailed results of ANOVAs for each brain region are summarized in Supplementary Table S3.

### 3.7. Parvalbumin Expression in Inhibitory Neurons in the Brain

Several cortical regions in the anterior forebrain of PN14 pups were examined by immunohistochemistry for the calcium-binding protein Pvalb, which is expressed in a subset of inhibitory neurons.

**Figure 7:**
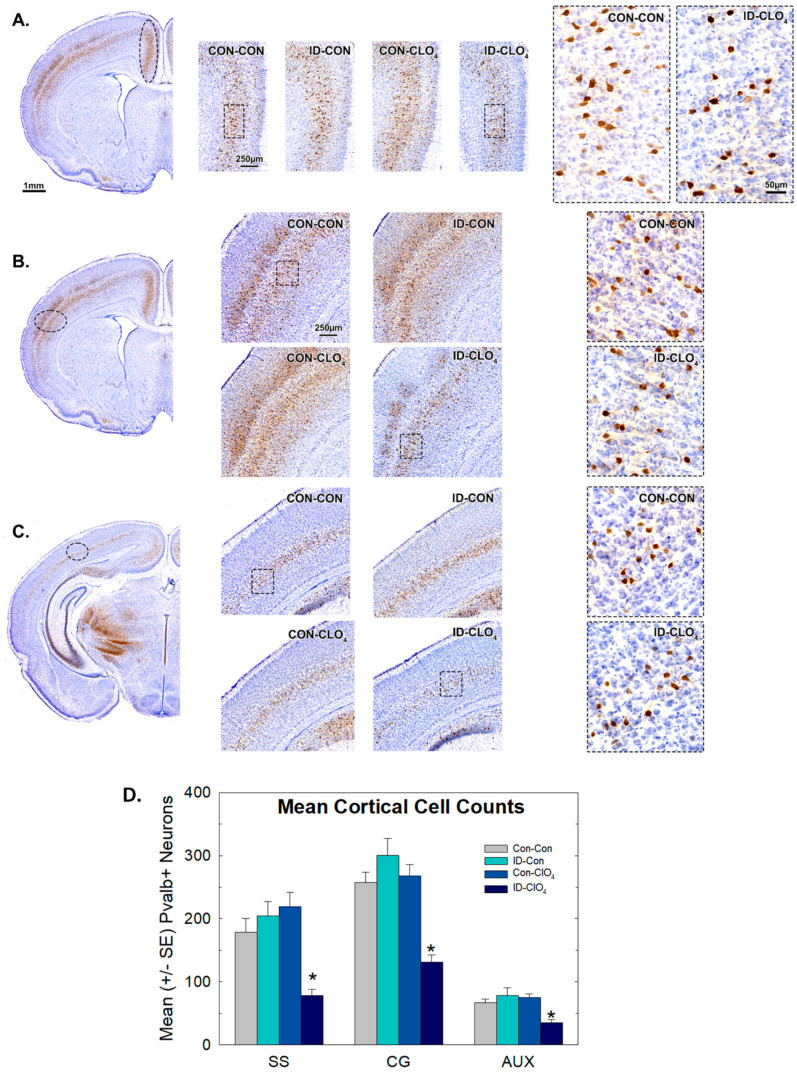
Immunoreactivity for Pvalb^+^ neurons in pup neocortex. Fewer Pvalb^+^ cells were present in three cortical regions of ID-ClO_4_-exposed pups. (**A**) Representative Pvalb immuno reactivity images from one animal from each treatment condition for (**A**) cingulate, CC; (**B**) somatosensory, SS; and (**C**) auditory, Aux cortex. (**B**) Regions in the anterior and posterior cortex selected for cell counts are indicated by dotted ovals on images on the far left. High-magnification images of Pvalb+ cells are shown on the far right for the area indicated by the bounding box in the sections of the middle panel. (**D**) Group mean (+/−SE) cell counts in each treatment condition are presented as mean per litter, collapsed across sex. Mean contrasts supported a significant reduction in Pvalb^+^ cell numbers that was restricted to the ID-ClO_4_ group in all three cortical regions. Sample size n = 10–12 litters/treatment condition. * Dunnett’s *t*-test, significantly different from Con-Con using α = 0.05.

On visual inspection, Pvalb^+^ cells were clearly observable in the somatosensory and cingulate cortices in all groups, but with a more diminished presence in animals exposed to ID-ClO_4_ (Figure 7A,B). Pvalb^+^ staining was not visibly altered by ClO_4_ or ID alone, and no sex differences were observed. Although immunostaining for Pvalb^+^ cells was less prominent in the posterior and ventral portions of the brain at this age, irrespective of treatment, a similar treatment-related reduction in expression was also seen in the more posteriorly located auditory cortex (Figure 7C). These qualitative assessments were verified quantitatively by determining the mean number of Pvalb^+^ cells in these cortical regions (8–12 males or 7–10 females/treatment group). An overall ANOVA did not reveal a significant main effect of sex or a sex and treatment interaction (*p* = 0.33 or higher), so quantitative data were summarized as litter means, collapsed across sex (Figure 7D). ANOVAs indicated significant main effects of treatment in somatosensory [F_(7,66)_ = 7.9, *p* < 0.0001], cingulate [F_(7,66)_ = 11.31, *p* < 0.0001], and auditory cortices [F_(3,38)_ = 7.90, *p* = 0.0003] and Dunnett’s confirmed significant reductions in the ID-ClO_4_ group relative to the Con-Con group.

**Figure 8:**
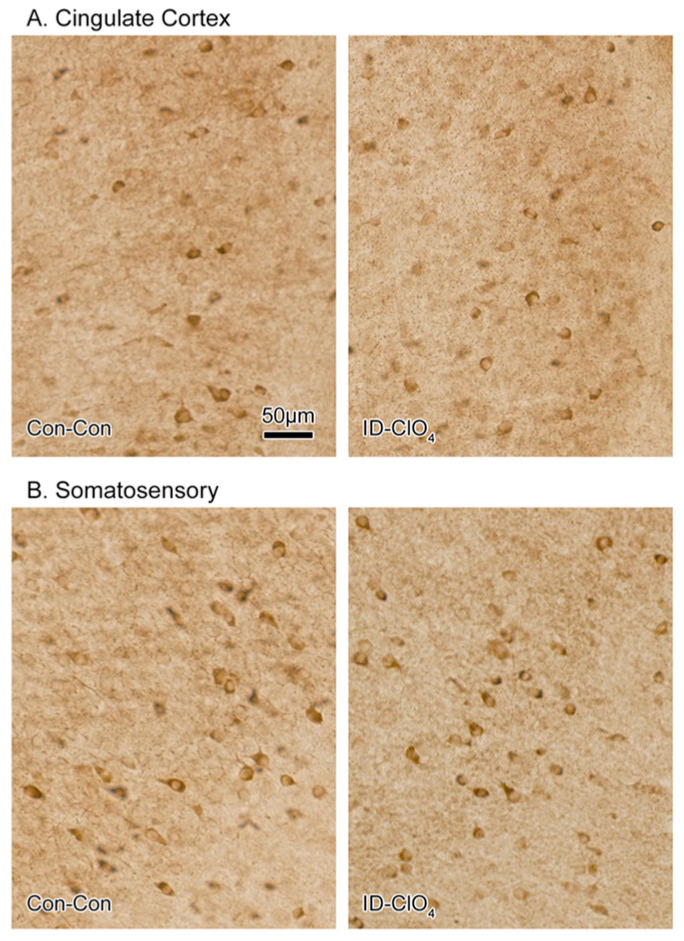
Immunostaining for the GABA synthesis enzyme, GAD-67, was conducted on a series of sections from Con-Con and ID-ClO_4_ (n = 5/treatment condition). No difference was detected in GAD-67 immunostaining in the (**A**) cingulate or (**B**) somatosensory cortex based on qualitative assessments by 4 independent observers blind to the treatment condition.

To determine if lower Pvalb+ interneuron counts in the cortex reflected either a general loss of inhibitory neurons in these regions or a loss of expression of this protein, we performed immunohistochemistry for GAD-67, a synthesis enzyme present in almost all GABA neurons. No difference in GAD-67 staining was seen between Con-Con and ID-ClO_4_ animals in the cingulate or the somatosensory cortex (Figure 8).

### 3.8. Behavioral Measures in Adult Offspring

Two neurobehavioral assessments were performed in adult male and female offspring 1–2 months after the cessation of exposure and return to iodine-sufficient diets. The same animals were assessed in both paradigms.

#### 3.8.1. Distract Trace Fear Conditioning

**Figure 9:**
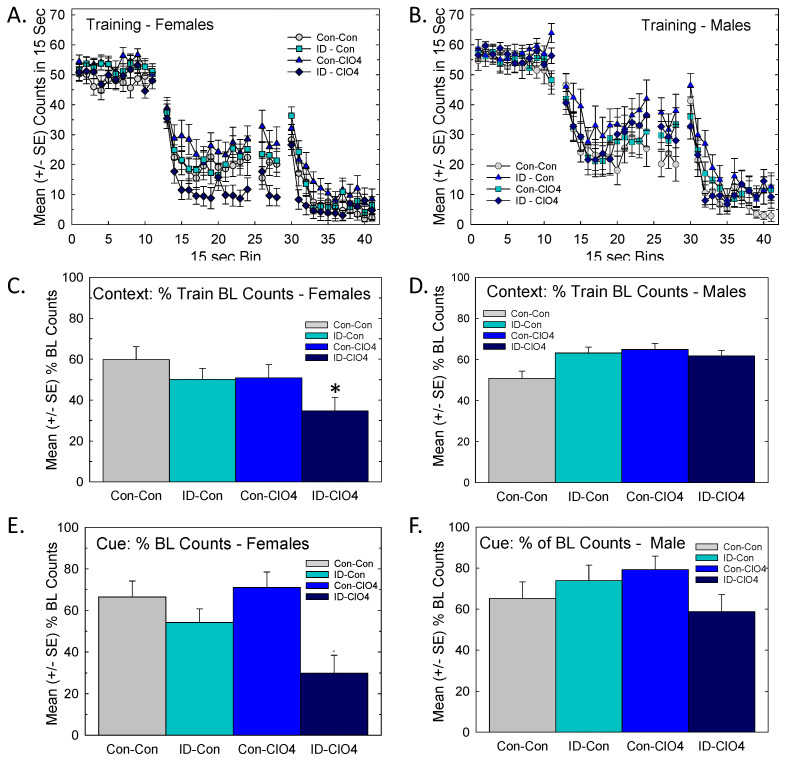
Trace fear conditioning was altered in females (**left** panels) but not males (**right** panels). Trace fear conditioning was altered in female (**left** panels) but not male (**right** panels) offspring. (**A**) Training Phase: Mean activity counts in 15-s bins during trace fear acquisition training did not differ among treatment conditions. All groups learned equally well. (**B**) Context Learning: In a test of conditioning to context 24 h after training, the suppression of activity relative to training baseline was seen in females (**left**) of the ID-ClO_4_ group, while male activity levels (**right**) were unchanged relative to Con-Con. (**C**) Cue Learning: A similar but less robust pattern was seen in cue learning in females (**E**). No significant difference in cue learning in females or cue or context learning was observed in males (**D**,**F**). Litter was used as a random variable and sample sizes were 17, 18, 26, and 19 for female offspring and 15, 20, 12, and 17 for male offspring in groups Con-Con, ID-Con, Con-ClO_4_, and ID-ClO_4_, respectively. * Contrast relative to Con-Con significant using α = 0.05, Bonferroni-adjusted for multiple comparisons.

No group differences were evident in either sex in baseline activity patterns or distract trace fear acquisition during fear training (Figure 9A,B). Context learning assessed the following day showed no difference in males (Figure 9D), while females exhibited significantly lower activity counts, most notably in the ID-ClO_4_ group (Figure 9C). An ANOVA revealed a significant dose by sex interaction for context conditioning [F_(3,111)_ = 3.86, *p* = 0.0113]. Differences in the least-squares means with Bonferroni adjustment for multiple comparisons supported a greater suppression of activity in females of the ID-ClO_4_ condition relative to the Con-Con group (*p* = 0.0267). Increased activity levels were taken as evidence of impaired learning, whereas a suppression of activity observed in females could represent either enhanced learning or increased fear response.

A very similar pattern was evident in conditioning to cue, where females (Figure 9E), but not males (Figure 9F), of the co-exposed group showed suppression of activity during the trace interval following the delivery of the tone/light CS. The analysis of cue learning returned a significant main effect of sex [F(_1,114)_ = 5.65, *p* = 0.0191] and a marginal effect of dose [F_(3,38.4)_ = 2.48, *p* = 0.0759] but no effect of dose and sex interaction [F_(3,113)_ = 1.15, *p* = 0.3324]. Effects on cue learning in females were not significant when Bonferroni adjustment for multiple comparisons was applied (Figure 9E). As in context learning, no significant effect was found for cue learning in males (Figure 9F).

#### 3.8.2. Acoustic Startle and Prepulse Inhibition

**Figure 10:**
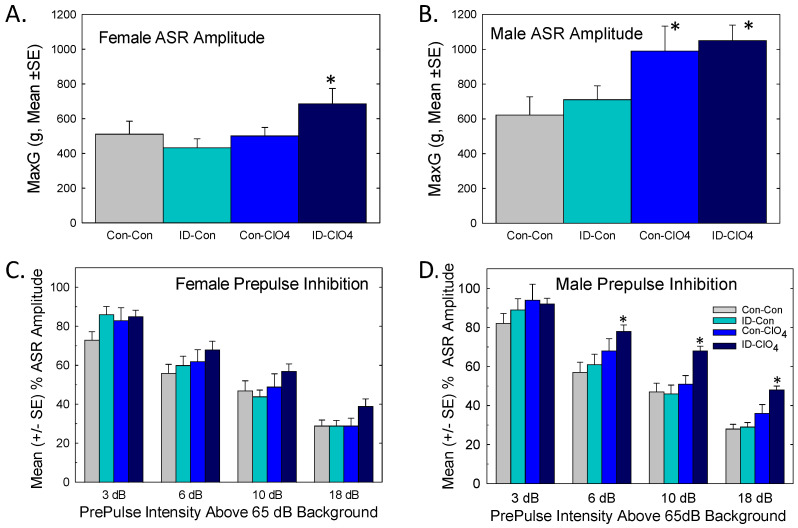
Acoustic startle response (ASR) and prepulse inhibition (PPI). Mean (+/−SE) baseline ASR was increased in (**A**) adult female offspring from the Con-ClO_4_ treatment condition and (**B**) male offspring from both the Con-ClO_4_ and the ID-ClO_4_ conditions. (**C**) Prepulse inhibition of the ASR was increased as a function of prepulse intensity, but no treatment-related differences were evident in females. (**D**) Adult male offspring of the ID-ClO_4_ group exhibited a diminished suppression of the ASR at the 6, 10, and 18 dB levels. Litter was used as a random variable in the statistical model and sample sizes varied from 15 to 26/sex/treatment group and 10 to 13 litters/treatment group. * Contrast relative to Con-Con significant using α = 0.05, Bonferroni-adjusted for multiple comparisons.

Baseline ASR was assessed prior to PPI. As expected, the basal ASR was smaller in females (Figure 10A) than males (Figure 10B), largely reflective of differences in muscle mass and body weight, observations supported by a significant main effect of sex [F_(1,92.6)_ = 50.8, *p* < 0.0001]. Perchlorate exposure increased ASR relative to the control in male but not female offspring in the Con-ClO_4_ group. The augmentation of ASR was also present in males and females from the co-exposed group [dose F_(1,35.7)_ = 9.15, *p* = 0.0046]. Habituation of the ASR over the six-trial blocks prior to PPI testing was comparable across treatment groups in both males and females (no significant interactions of dose and diet or sex and dose and diet were observed).

In the PPI sensory gaiting test, brief prepulses of low-intensity sound were presented in advance of the startle stimulus against a backdrop of background noise. The results of PPI are summarized in Figure 10C,D. An overall ANOVA revealed significant main effects of sex [F_(1,102)_ = 7.2, *p* = 0.0085], where males but not females were affected, and PP intensity [F_(3,395)_ = 355, *p* < 0.0001], with an intensity-dependent reduction in ASR in the presence of a prepulse. A significant treatment and sex interaction [F_(1,102)_ = 4.67, *p* = 0.033] supported the treatment effect on PPI and was limited to males. A significant dose and diet and PP intensity interaction was found [F_(3,395)_ = 3.86, *p* = 0.0097]. Differences in the least-squares means indicated that the ID-ClO_4_ group had significantly lower PPI than the Con-Con group at the three highest PP intensities (*p* = 0.014 or lower). No effects on PPI were evident in perchlorate-alone or ID-alone males or females from any treatment condition. Deficits in PPI could not be readily explained by minor differences in mean body weight across groups at the time of testing. Neither were they easily attributed to an augmented baseline ASR as PPI differences were not detected in females or males exposed to perchlorate alone, both of which exhibited augmented baseline startle responses.

## 4. Discussion

We report an exacerbation of the effects of developmental exposure to perchlorate on the brain by concomitant dietary iodine deficiency. Consistent with previous work from our laboratory, we observed modest reductions in serum T4 in dams and no effects on the thyroid hormones of pups in response to maternal exposure to a 300 ppm dose of perchlorate [20,21,22] or at this level of iodine deficiency [26]. However, when ID was coupled with perchlorate, reductions in serum hormones and transcriptional alterations in the thyroid gland and brain were detected. These observations were accompanied by two downstream adverse consequences in the offspring: reductions in the number of Pvalb^+^ interneurons in the neonate and persistent neurobehavioral impairments in the adult.

Despite the presence of perchlorate in sera and milk bands of both groups of pups receiving perchlorate, serum hormone deficits were restricted to the group exposed to perchlorate under ID conditions. Consistent with previous reports, *Nis* expression in the newborn thyroid gland increased >20-fold with perchlorate exposure, and the concentrations of perchlorate in the serum declined over the neonatal period [22,24]. In a previous study, we concluded that limited lactational exposure and the compensatory augmentation of *Nis* expression were sufficient to regain ‘euthyroid’ conditions in the pups [22]. In the present study, no change in *Nis* expression was evident in ID pups, and, consistent with previous reports, both ID- and perchlorate-exposed pups maintained serum T4 comparable to controls [20,22,24,26]. In contrast, here, we report that in the co-exposed group, despite a >30-fold ‘compensatory’ upregulation of gland *Nis* expression at birth, serum TH was significantly suppressed.

In the brain, evidence of altered TH action was observed. In all three brain regions examined in the PN14 pup, the downregulation of a number of TH-responsive gene targets was present and largely restricted to the co-exposed group. A greater number of TH-responsive transcripts was altered in the cortex (seven of nine investigated) than in the hippocampus or cerebellum, an observation consistent with previous findings with the synthesis inhibitor propylthiouracil (PTU) [51]. There was little evidence of changes in gene expression in the ID-Con or Con-ClO_4_ groups, also consistent with a recent report at this dose of perchlorate [22].

One of the primary findings of the present study was the reduction in Pvalb^+^-expressing interneurons in the brains of neonates. Despite being far outnumbered by excitatory neurons, the rich synaptic plexus of inhibitory interneurons accounts for a large percentage of all cortical synapses [52]. Disruptions in the timing and distribution of this population of cells have been implicated in the origins of a variety of neurodevelopmental disorders [52,53,54,55,56,57]. Mouse models of neurodevelopmental disorders induced by genetic mutations clearly demonstrate the critical role of Pvalb^+^ interneurons in the shaping of neuronal circuitry in the developing brain [53,54,55,56]. Reductions in the number of Pvalb^+^ neurons can disrupt the formation of neural circuits, upsetting the delicate balance of excitation and inhibition during critical periods of brain development [52,53,54,55,56,57].

The vulnerability of Pvalb^+^-neurons appears to be a common response to developmental disruption of the thyroid system. It is seen in the brains of humans suffering from mutations of the TH transporter MCT8 and in mice with genetic modifications of TH transporters, selenoproteins, deiodinases, or TH receptors (see reviews by [58,59,60]). Reductions in Pvalb expression are also seen with developmental exposure to the TH synthesis inhibitors PTU and methimazole (MMI) [61,62]. As in PTU and MMI models, we observed a reduction in Pvalb^+^ neurons in the absence of change in GAD-67 immunoreactivity, a marker for all GABA neurons [61,62]. No difference in GAD-67 staining indicated that the full complement of GABAergic neurons remained intact under these conditions of TH compromise. This observation in the presence of a selective reduction in immunostaining for Pvalb^+^ suggested that these GABA-expressing cells either failed to produce this protein or the timing of its production was delayed [58,61]. Alternatively, as all inhibitory GABAergic interneurons are derived from the ganglionic eminences during fetal brain development, it is possible that TH-dependent signaling pathways may directly or indirectly drive cell fate determination at this early embryonic stage [59,63]. However, the lack of effect of Pvalb expression when ID or perchlorate was delivered in isolation, both conditions where fetal thyroid hormone reductions were present [22,26], favors an interpretation of interference with the postnatal influence of TH on Pvalb expression over a prenatal action on cell fate specificity. Observations from primarily postnatal MMI treatment, cross-fostering manipulations, and reversal of the Pvalb+ deficiencies with hormone replacement in the PTU model also support a primarily postnatal influence of TH insufficiency on this population of Pvalb-expressing interneurons [61,62].

TH insufficiency and the accompanying loss of Pvalb+ interneurons may have long-lasting consequences on brain function, including impaired neurotransmission, seizures, and neurobehavioral deficits [58,59]. We examined distract trace fear conditioning, a paradigm that introduced a higher cognitive and attentional demand into a simple fear-motivated associative task [64]. Fear learning requires activation of the amygdala, while the incorporation of a distractor stimulus and a trace element during training and the evaluation of contextual learning engages the cortex and hippocampus [64,65,66,67,68]. Relative to controls, females of the co-exposed group exhibited reductions in activity in both context and cue testing, suggestive of ‘enhanced’ learning. Alternatively, this pattern may reflect an exaggerated emotional fear response to contextual and explicit CS cues previously associated with shock. Sex differences in learned fear are well documented in humans and other animals and have been attributed to functional differences between the sexes in the medial prefrontal cortex [69,70,71,72,73]. Augmented fear and anxiety restricted to female offspring suggests a sex-dependent disruption of amygdala-mediated fear or a medial prefrontal dysfunction over the hippocampally mediated memorial components of the trace fear paradigm. In previous studies of developmental TH insufficiency induced by PTU, trace fear learning was impaired in males, but female offspring were not affected [41], and Barez-Lopez et al. [74] reported augmented fear responses in male deiodinase-2 knock-out mice; females were not tested. As such, the current findings of differential responding in a fear-motivated task in females with co-exposure to ID and perchlorate await replication, and the role of developmental TH insufficiency in trace fear learning requires further study.

Sex-dependent behavioral effects were also observed in PPI of the ASR, but, in this case, males were more affected than females. Baseline startle responses were elevated in both male and female adult offspring of the co-exposed group. Males of the perchlorate-alone exposure group also exhibited an enhanced startle response. As expected for PPI, low-intensity prepulses delivered in advance of the loud startle-eliciting stimulus produced intensity-dependent reductions in the startle response in both male and female offspring from all groups. However, at the three highest prepulse levels, reductions in the relative suppression were apparent in males from the co-exposed group. PPI is impaired in human and animal models of neurological diseases including autism, schizophrenia, obsessive–compulsive disorder, epilepsy, and Tourettes’s syndrome [43,44,75]. The pharmacological enhancement of GABAergic inhibition is used as a therapeutic strategy for these disorders [43,44]. Although it is not possible to directly link these behavioral impairments to the reductions in Pvalb+ interneurons observed in this study, it is noteworthy that PPI was reduced by the pharmacological blockade of GABAergic neurotransmission in male rats [76], in Pvalb-deficient male mice [77], and with the selective inhibition of Pvalb-expressing neurons in the ventral hippocampus of male mice [78]. Furthermore, the trophic properties of estrogen appear to be protective of behavior impairment in PPI and may contribute to the sex-specific impairments evident in PPI in the present study [79].

### 4.1. Impact and Relevance

The concentration of perchlorate examined in this study reduced maternal serum T4 concentrations by ~50% but was without effects on serum T4 in neonatal offspring. In the pregnant rats, the 300 ppm dose level equated to a perchlorate intake of ~23 mg/kg/day [22,80]. As with most animal studies, this dose of perchlorate exceeded the estimated average daily intake in humans, which ranges from 0.09 to 0.48 µg/kg/day based on age [81,82]. However, a recent rodent study revealed that a much lower concentration of maternal perchlorate exposure (1 ppm, equivalent to a perchlorate intake of ~0.07–0.09 mg/kg/day in the dam) is sufficient to reduce TH synthesis in the fetal thyroid gland [21,80]. Reductions in TH synthesis in the gland were not reflected in serum TH reductions at this fetal life stage, suggesting that the observed stimulation by TSH and the upregulation of *Nis* were able to compensate under iodine *sufficient* conditions. As shown here, however, ID shifted the potency curve for perchlorate to the left. The low dose effects on gland synthesis at 1 ppm reported by Gilbert et al. [21], when combined with ID, may be sufficient to overwhelm the compensatory response of the fetal thyroid gland, reduce serum TH levels, and negatively impact brain development in rats. It is difficult to extrapolate the rat TH perturbations to humans because of the lengthy exposure time required to deplete the thyroid gland of iodide in adult humans. However, like rats, the capacity for iodine storage in humans is much lower in the fetal relative to the maternal thyroid gland. These effects on the fetal thyroid gland observed at low doses of perchlorate [21] and exacerbation of the negative impact of perchlorate on the developing brain when delivered under ID conditions reported here and elsewhere [24] should be considered when evaluating the potential of perchlorate to induce developmental neurotoxicity in humans.

### 4.2. Summary and Conclusions

The results from this study support previous findings in rodents where both thyroid and brain effects of perchlorate and ID, prominent in the fetus, were not maintained in the neonate when each was delivered in isolation. Exposure dynamics and absorption/distribution/metabolism/elimination changes that occur at birth along with the resilience of the thyroid axis of newborn pups appear to limit the impact of ID or ClO_4_ on TH in the serum and brain of neonates [22,26]. The findings also support recently reported effects on the absence of a specific TH-dependent structural defect, a periventricular heterotopia, in the brains of ID animals [24]. While heterotopia were detectable in the brains of ClO_4_-treated animals, they were quite small. However, parallelling the findings of the current study, this structural defect was very pronounced when ID was paired with ClO_4_ [24]. Importantly, using this same cohort of animals, here, we extended these observations to reveal reductions in numbers of Pvalb+ cortical inhibitory interneurons and neurobehavioral impairments. Disruptions in the functionality, maturation, and timing of Pvalb+ interneurons can impair the formation of neural networks throughout the developing brain and contribute to a diverse set of neurodevelopmental disorders [52,83,84,85,86,87]. Together with our previous reports, these observations underscore the critical need to consider additional non-chemical stressors in the determination of hazards and risks posed by environmental contaminants that affect the thyroid system and developing brain.

## Data Availability

All data reported are publicly available on ScienceHub.

## Supplementary Materials

The following are available online at https://www.mdpi.com/article/10.3390/toxics12120842/s1, Supplementary Table S1: Target and references genes for thyroid and brain gene expression; Supplementary Table S2: Summary statistics for gene expression in thyroid gland of pups on PN0, PN2 and PN14 and in dams on PN21; Supplementary Table S3: Summary statistics for gene expression in cortex, hippocampus, cerebellum of PN14 rat pup brains.
