# Supplementary material for: Iodine Deficiency Exacerbates Thyroidal and Neurological Effects of Developmental Perchlorate Exposure in the Neonatal and Adult Rat
Source: Toxics. 2024 Nov 23;12(12):842. doi: 10.3390/toxics12120842 (PMC11679215; doi:10.3390/toxics12120842)
Supplement: Supplementary file 1 [file toxics-12-00842-s001.zip › toxics-3223266-supplementary.pdf]

Supplementary Table S1: Target and references genes for thyroid and brain gene expression

**Thyroid Gland Transcripts**	*Gene Symbol*	Gene Name	Gene ID
	Slc5a5	Sodium iodine symporter - Nis	Rn00583900_m1
	Dio1	Deiodinase 1	Rn00572183_m1
	Nkx2.1	NK2 Homeobox 1, transcription factor	Rn01512482_m1
	Pax8	Paired Box 8, transcription factor	Rn00579743_m1
	Tpo	Thyroperoxidase	Rn00571159_m1
	Tg	Thyroglobulin	Rn00667257_g1
	TshR	Thyroid stimulating hormone receptor	Rn00563612_m1
**Brain Transcripts PN14**			
Cortex, Hippocampus	*Agt*	Angiotensinogen	Rn00593114_m1
Hippocampus, Cerebellum	$Bdnf_4$	Brain-derived neurotrophic factor- 4	Rn01484927_m1
Cerebellum	*Camk4*	Calcium/calmodulin-dependent protein kinase 4	Rn00664802_m1
Cortex, Hippocampus, Cerebellum	*Col11a2*	Collagen type XI α 2 Chain	Rn01428773_g1
Cortex	*Gjb6*	Gap junction protein, beta 6	Rn02042582_s1
Cortex	*Hopx*	HOP Homeobox	Rn00592446_m1
Cortex, Hippocampus, Cerebellum	*Hr*	Hairless, HR Lysine Demethylase and Nuclear Receptor Corepressor	Rn00577605_m1
Cortex	*Itih3*	Inter-α-trypsin inhibitor heavy chain 3	Rn00569293_m1
Cortex, Hippocampus, Cerebellum	*Klf9 (Bteb1)*	Kruppel-like factor 9 (Basic transcription element binding protein)	Rn00589498_m1
Hippocampus, Cerebellum	*Mag*	Myelin-associated glycoprotein	Rn02586362_m1
Cerebellum	*Mog*	Myelin oligodendrocyte glycoprotein	Rn00575354_m1
Hippocampus	*Ngf*	Nerve growth factor	Rn01533872_m1
Cerebellum	*Pcp2*	Purkinje cell protein 2	Rn01403231_g1
Cortex	*Pnoc*	Prepronociceptin	Rn01637101_m1
Cortex, Hippocampus, Cerebellum	*Pvalb*	Parvalbumin	Rn00574541_m1
**Reference Genes**			
Thyroid, Cortex, Cerebellum	*β2m*	β-2microglobulin	Rn00560865_m1
Hippocampus	*β -actin*	β-actin	Rn00667869_m1

Supplementary Table S2: Summary statistics for gene expression in thyroid gland of pups on PN0, PN2 and PN14 and in dams on PN21. **reach criteria of FC>1.5, α<0.02. Dunnett's t-test, significantly different from Con-Con using α=0.05 are shown in Figure 4.

Slc52a (Nis)	**Overall ANOVA**<br>**df**	**f**	**p**	**StepDown Oneway ANOVAs by Age**			
age	2			**PN0**	**PN2**	**PN14**	**Dams**
treatment	3	24.44	<0.0001				
age*treatment	6	22.45	<0.0001	F(3,19)=10.14, p=0.0003	F(3,20)=20.85, p<0.0001	F(3,28)=41.36, p<0.0001	F(3,28)=5.57, p=0.0040
Dio2		8.1	<0.0001	**	**	**	**
age	2						
treatment	3	2.15	=0.1249				
age*treatment	6	4.53	=0.0059	F(3,19)=7.71, p=0.0021	F(3,20)=0.29, p=0.8310	F(3,28)=10.25, p<0.0001	F(3,28)=1.61, p=0.2094
Nkx2.1		5.05	=0.0003	**	n.s	**	n.s
age	2						
treatment	3	10.8	<0.0001				
age*treatment	6	16.44	<0.0001	F(3,19)=7.15 p=0.0021	F(3,20)=7.04, p=0.0020	F(3,28)=15.14, p=0.0059	F(3,28)=1.22, p=0.3200
Pax8		1.12	=0.3581	**	**	**	n.s
age	2						
treatment	3	16.9	<0.0001				
age*treatment	6	10.67	<0.0001	F(3,19)=16.03, p<0.0001	F(3,20)=0.95, p=0.4333	F(3,28)=4.27, p=0.0133	F(3,28)=13.41, p<0.0001
Tg		1.6	=0.1597	**	n.s	**	**
age	2						
treatment	3	0.71	=0.4955				
age*treatment	6	22.59	<0.0001	F(3,19)=7.48, p=0.0017	F(3,20)=8.46, p=0.0008	F(3,28)=8.02, p=0.0005	F(3,28)=3.05, p=0.0450
Tpo		0.24	=0.9631	**	**	**	n.s
age	2						
treatment	3	9.24	=0.0003				
age*treatment	6	11.15	<0.0001	F(3,19)=3.30, p=0.0426	F(3,20)=3.94, p=0.0232	F(3,28)=8.73, p=0.0003	F(3,28)=4.18, p=0.0145
TshR		2.27	=0.0473	n.s.	n.s.	**	**
age	2						

treatment	3	19.75	<0.0001				
age*treatment	6	32.51	<0.0001	F(3,19)=24.07, p<0.0001	F(3,20)=13.88, p<0.0001	F(3,28)=6.79, p<0.0001	F(3,28)=9.62, p=0.0002

Supplementary Table S3: Summary statistics for gene expression in cortex, hippocampus, cerebellum of PN14 rat pup brains. Dunnett's t-test, significantly different from Con-Con using α=0.05 are shown in Figure 6.

Cortex	Target	ANOVA	Pass?<br>FC>1.25 α<0.02	Group Differences detected by Dunnett's?
	Agt	F(3,39)=21.93, p<0.0001	yes	yes
	Col11a2	F(3,39)=5.80, p=0.0022	yes	yes
	Gjb6	F(3,39)=12.10, p<0.0001	yes	yes
	Hop	F(3,39)=9.57, p=0.0001	yes	yes
	Hr	F(3,39)=8.50, p=0.0002	yes	yes
	Itih3	F(3,39)=16.44, p<0.0001	yes	yes
	Klf9	F(3,39)=3.75 p=0.0185	yes	no
	Pvalb	F(3,39)=12.67, p<0.0001	yes	yes
	Pnoc	F(3,39)=1.55, p=0.2162	no	n/a

Hippocampus	*Agt*	F(3,37)=5.65, p=0.0027	yes	no
	Bdnf4	F(3,37)=1.58, p=0.2107	no	n/a
	Coll11a2	F(3,37)=5.80, p=0.0023	yes	yes
	Hr	F(3,37)=11.12, p=0.0004	yes	yes
	Klf9	F(3,37)=3.24, p=0.0329	no	n/a
	Mag	F(3,37)=5.93, p=0.0021	yes	no
	Ngf	F(3,37)=1.68, p=0.1889	no	n/a
	Pvalb	F(3,37)=4.48, p=0.0088	yes	no

Cerebellum	*Bdnf4*	F(3,16)=0.47, p=0.7076	no	n/a
	Camk4	F(3,16)=4.20, p=0.0226	no	n/a
	Col11a2	F(3,16)=9.98, p=0.0006	yes	yes
	Hr	F(3,16)=8.03, p=0.0017	yes	yes
	Klf9	F(3,16)=9.22, p=0.0009	yes	yes
	Mag	F(3,16)=2.25, p=0.1241	no	n/a
	Mog	F(3,16)=3.21, p=0.0533	no	n/a
	Pvalb	F(3,16)=8.74, p=0.0014	yes	yes

	Pcp2	F(3,16)=2.05, p=0.1506	no	n/a
